# Effects of light intensity on growth and lipid production in microalgae grown in wastewater

**DOI:** 10.1186/s13068-019-1646-x

**Published:** 2020-01-07

**Authors:** Jean Claude Nzayisenga, Xavier Farge, Sophia Leticia Groll, Anita Sellstedt

**Affiliations:** 1 Department of Plant Physiology, UPSC, Umea University, 90187 Umea, Sweden; 2 Present Address: Graduate School of Biotechnology of Strasbourg (ESBS), Unistra, 67400 Illkirch-Graffenstaden, France

**Keywords:** Biodiesel, Biorefinery, FTIR, Light intensity, Lipids, Microalgae

## Abstract

**Background:**

Cultivation of microalgae in wastewater could significantly contribute to wastewater treatment, biodiesel production, and thus the transition to renewable energy. However, more information on effects of environmental factors, including light intensity, on their growth and composition (particularly fatty acid contents) is required. Therefore, we investigated the biomass and fatty acid production of four microalgal species, isolated in the Northern hemisphere and grown at three light intensities (50, 150 and 300 μE m^−2^ s^−1^).

**Results:**

Increases in light intensities resulted in higher biomass of all four species and, importantly, raised fatty acid contents of both *Desmodesmus* sp. and *Scenedesmus obliquus*. Fourier-transform IR spectrometry analysis showed that the increases in fatty acid content were associated with reductions in protein, but not carbohydrate, contents. Assessment of fatty acid composition revealed that increasing light intensity led to higher and lower contents of oleic (18:1) and linolenic (18:3) acids, respectively. The microalgae consumed more than 75% of the nitrogen and phosphorus present in the wastewater used as growth medium.

**Conclusion:**

The results show the importance of optimizing light intensities to improve fatty acid production by microalgae and their quality as sources of biodiesel. In addition, increase in fatty acid content is associated with decrease in protein content.

## Background

The increasing demand for energy and the negative environmental impacts of fossil fuel use are prompting global searches for renewable and clean fuels [1]. Many researchers are studying microalgae-based biofuels as promising candidates to replace fossil fuels. Microalgae are a group of photosynthetic organisms that can produce organic molecules including lipids, which can be used to generate biodiesel [2]. To get a viable fuel, the growth of algae for biodiesel production should be cost-effective. Algal growth relies mostly on two nutrients: nitrogen and phosphorus [3]. Levels of these nutrients in wastewater, such as municipal wastewater, are often too high for safe environmental release, but they are expensive to remove [2, 4]. Therefore, using municipal wastewater to grow algae may provide an efficient means to both clean the wastewater cheaply and generate biofuel.

Generally, increases in light intensity increase microalgal growth up to a photoinhibitory threshold, but both the strength of this effect and the threshold vary among species [5, 6]. Light intensity also influences microalgal lipid production, which is of particular interest because lipids are the sources of biodiesel (as described below). However, increases in light intensity reduce lipid contents of some species [7], but promote or have no effect on lipid production in others [8, 9]. Therefore, it is important to study the effects of light intensity on lipid production, on a species-by-species basis.

Microalgal biomass is mostly composed of lipids, carbohydrates, and proteins [10]. Therefore, if lipid contents increase there should be corresponding reductions in contents of carbohydrates, proteins, or both. Nitrogen starvation often reportedly leads to an increase in lipids and a decrease in carbohydrate content [11–13]. However, little is known about how light intensity affects the biochemical composition of microalgae, apart from the variable effects on lipid production mentioned above. Therefore, it is important to determine how the production of all three biochemical components changes with light intensity in order to optimize microalgal lipid production to generate biodiesel.

Biodiesel is produced from neutral lipids, primarily in the form of triacylglycerols, which contain three fatty acids linked by glycerol. Transmethylation of triacylglycerols results in fatty acid methyl esters (FAMEs), which make up biodiesel, and glycerol as a byproduct [11, 12]. Fatty acid composition is an important factor to consider for the successful generation of biodiesel from algae (or any other biomaterial). For example, biodiesel with high amounts of polyunsaturated fatty acids can be readily oxidized due to the presence of double bonds in the fatty acid chains. In addition, biodiesel with high amounts of saturated fatty acids can solidify. Light may reportedly affect fatty acid composition and, therefore, biodiesel properties [13, 14]. However, few studies have focused on the effects of light intensity on fatty acid composition.

Therefore, we examined effects of three light intensities (50, 150 and 300 µmol m^−2^ s^−1^) on the biomass of four species of microalgae isolated in the Northern hemisphere and grown in wastewater. Using Fourier-transform IR spectrometry (FTIRS) analysis, we also examined the relative abundance of lipids, carbohydrate and proteins under each of the treatments. We also evaluated fatty acids content and profile using gas chromatography.

## Results and discussion

### Effects of light intensity on biomass production

**Fig. 1:**
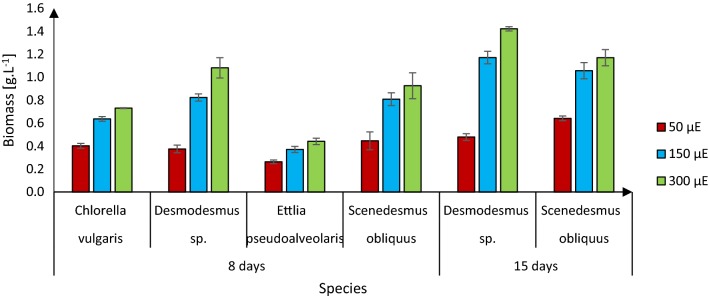
Biomass of the four microalgal strains after growth for indicated times under indicated light intensities: mean ± standard deviation (*n* = 3 from three separate experiments)

The highest biomass we recorded in our cultivations of the four species of microalgae for 8 days was 1.1 g/L, for *Desmodesmus* sp. grown at 300 μE m^−2^ s^−1^ light intensity (Fig. 1). The biomass of *Desmodesmus* sp. cultures at this time point was positively correlated with the light intensity. However, increasing the light intensity from 150 to 300 μE m^−2^ s^−1^ did not significantly increase the biomass of *C. vulgaris* and *S. obliquus* cultures, which was ca. 0.6 and 0.8 g/L, respectively, at 150 μE m^−2^ s^−1^ light intensity after 8 days (Fig. 1). Thus, for those two species a light intensity of 150 μE m^−2^ s^−1^ was optimal for biomass production. The conclusion is that the threshold at the current condition for these two species is at 150 μE (Fig. 1). The results confirm general findings that up to a certain taxa-dependent saturating threshold light intensity limits growth of microalgae, and further increases would presumably have been photoinhibitory [5, 15]. To assess effects of a longer growth-period on biomass and fatty acid content, growth of the two species with the highest biomass, *Desmodesmus* sp. and *S. obliquus*, were cultivated for 15 days. After 15 days cultivation at 300 μE m^−2^ s^−1^, their biomass yields were 1.4 and 1.2 g/L, respectively (Fig. 1). Biomass yields of both species were still lowest at 50 μE m^−2^ s^−1^ (Fig. 1). According to these results, *Desmodesmus* yields the highest biomass, while *C. vulgaris* and *S. obliquus* grow optimally at 150 μE m^−2^ s^−1^. Thus, all three of those species are potential sources of biofuel, but *E. pseudoalveolaris* would not be a suitable source due to its low biomass production, at least under any of the conditions we applied.

### Effects of light intensity on fatty acid content

**Fig. 2:**
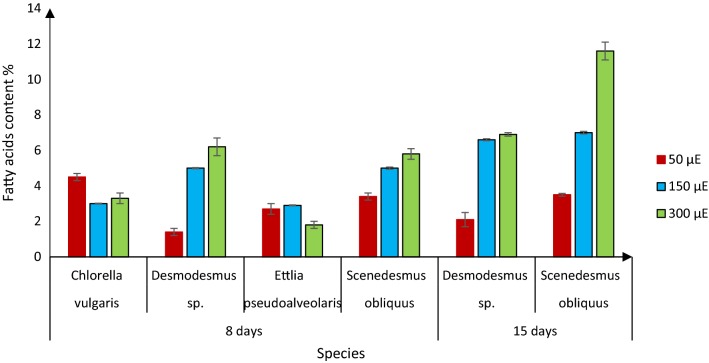
Fatty acid contents of the four microalgal strains after growth for indicated times under indicated light intensities: means ± standard deviations (*n* = 3 from three separate experiments)

The only types of lipids used for biodiesel production are fatty acids. While common gravimetric methods measure total lipid content, GC methods have the advantage of measuring contents of specific fatty acids [16]. Thus, we analysed the fatty acid content of each of the species cultivated under each of the three light intensities using a GC (with a FID) system. When grown at 300 μE m^−2^ s^−1^, *Desmodesmus* sp. had the highest content of fatty acids (6.2%), followed by *S. obliquus* (5.8%) at day 8 (Fig. 2). Moreover, fatty acid contents of these two species were positively correlated with the light intensity (Fig. 2). Our results are consistent with previous findings that algae grown at high light intensities often accumulate more lipids. For example, increasing light intensity from 55 to 110 μE m^−2^ s^−1^ has been found to increase lipid production by *S. abundans* [17], and several *Chlorella* species reportedly produce more lipids at a high light intensity (600 μE m^−2^ s^−1^) than at lower light intensities [18]. This may be at least partly because at high light intensities algae counter photooxidation by converting excess photoassimilates into fatty acids [19]. However, in some recent studies high light intensity reduced lipid contents of various microalgae, including marine strains of *Chlorella*, despite increasing their biomass [20, 21]. The cited authors suggested that the energy produced was used for cell division instead of being stored in the form of lipids [20, 21]. We also found that *C. vulgaris* and *E. pseudoalveolaris* had lower lipid contents when grown at 300 μE m^−2^ s^−1^ light than when grown at lower light intensities, despite increases in biomass (Fig. 2). Therefore, there may be differences in species’ mechanisms of responses to high light intensities, which result in either higher or lower lipid contents. We grew the two species with the highest biomass yields for 15 days. During the period between 8 and 15 days, fatty acid contents of *S. obliquus* growing at 300 μE m^−2^ s^−1^ light doubled, from 5.8 to 11.6%, but changed little at the 50 and 150 μE m^−2^ s^−1^ light intensities. In contrast, fatty acid contents of *Desmodesmus* sp. slightly increased during this period under all light intensities (Fig. 2). It has been suggested that increases in lipid production under high light intensities may be partly caused by starvation [22]. However, we found that nitrogen and phosphorus were still present in the medium after 15 days (Fig. 2).

### Effects of light intensity on biochemical composition

**Fig. 3:**
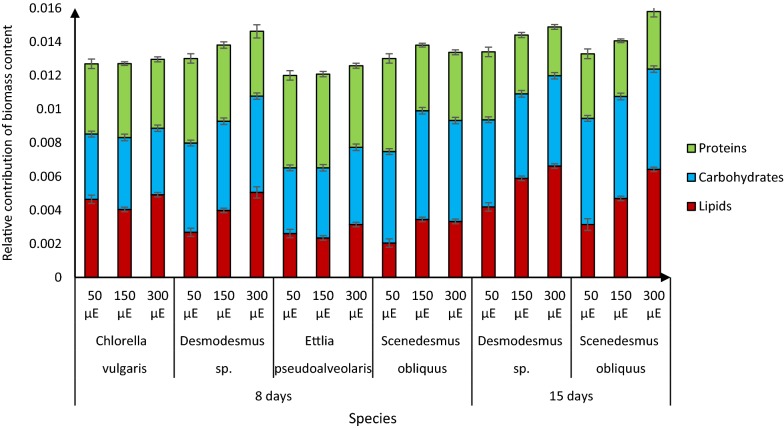
Biochemical composition of the four microalgal strains after growth for indicated times under indicated light intensities (50, 150, or 300 μE m^−2^ s^−1^): means ± standard deviations (*n* = 3 from two separate experiments)

Contrary to the effects of light on biomass and lipids, the impact of light intensity on proteins and carbohydrates has received little attention. To address this gap, we examined effects of light on the protein, carbohydrate and lipid contents of the microalgae using FTIRS methods, which reportedly provide results that correlate well with those obtained using standard extraction and analysis methods [23, 24]. With increases in light intensity, the fatty acid contents of *Desmodesmus* sp. and *S. obliquus* (grown for either 8 or 15 days) increased, their protein contents declined, and their carbohydrate contents did not significantly change (Fig. 3). Similarly, increases in lipid contents and reductions in protein contents of *Dunaliella tertiolecta* associated with increases in light intensity have been observed [25]. On the other hand, nitrogen starvation is reportedly associated with higher lipid, lower carbohydrate, and constant protein levels in *S. obliquus* and two *Chlorella* species [26–28]. For example, high lipid production in *Chrolella sorokiniana* under nitrogen starvation corresponded to starch degradation [27]. The hypothesis was that lipid and carbohydrate paths compete for a common carbon precursor [27, 29]. Thus, it has been suggested that blocking starch synthesis could increase lipid production [27]. However, our current results show that higher lipid content is linked to lower protein content, suggesting that lipid synthesis relied mostly on protein degradation or inhibition of protein synthesis. This is supported by He et al. [14], which showed that decrease of protein under increasing light intensity may be attributed to the consumption of nitrogen. It might also be that the provisions of carbon skeleton for amino acids and proteins synthesis might divert to serve as carbon and energy source for TAG biosynthesis [14]. In addition, although starch represents a more accessible form of carbon storage for plant cells than fatty acids, the energy recovery from fatty acid oxidation is greater than that of starch oxidation. When fatty acids are oxidized via the b-oxidation pathway and the citric acid cycle, the energy recovery is approximately 6.7 ATP equivalents per carbon for as an example palmitic acid [28].

It is indicated, that microalgae may have different mechanisms to synthesise fatty acids under high light intensities and/or nutrient starvation, which could affect either protein or carbohydrate content.

### Fatty acid composition under different light intensities

**Fig. 4:**
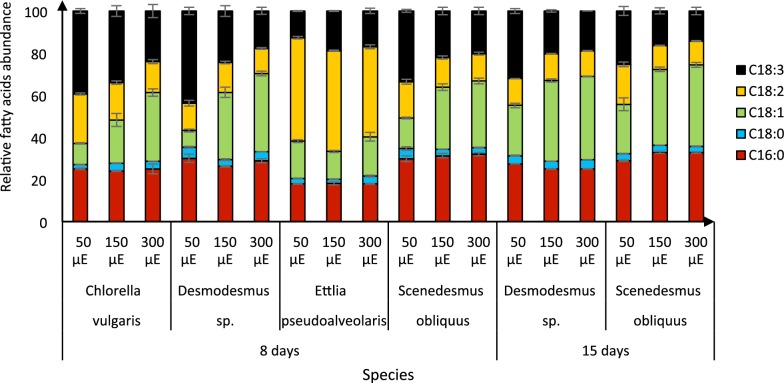
Fatty acid profiles of the four microalgae strains after growth for indicated times under indicated light intensities: means ± standard deviations (*n* = 3 from three separate experiments)

GC analysis of the fatty acid composition of the algae indicated that light intensity had similar effects on the fatty acid profile of all strains, except *E. pseudoalveolaris*, in which the fatty acid content was very similar under all three light intensities (Fig. 4). In the other three strains, 16:0 and 18:3 fatty acids were abundant, and 18:2 least abundant, at the lowest light intensity (Fig. 4). Increases in light intensity resulted in lower amounts of 18:3, and higher contents of 18:1, which became the most abundant fatty acid (Fig. 4). These results are consistent with previous findings that *C. protothecoides* had lower 18:3 and higher 18:1 contents when light intensity was increased from 35 to 420 μE m^−2^ s^−1^ [30]. Intriguingly, *E. pseudoalveolaris* had high 18:2 contents under all three light intensities, but it would be interesting to observe possible changes in its lipid composition at higher intensities. Biodiesel with a high content of polyunsaturated fatty acids such as 18:3 is prone to oxidation-dependent degradation [31]. By contrast, a high content of monounsaturated fatty acids such as 18:1, which are not susceptible to oxidation, increases biodiesel’s flow properties and reduces its solidification temperature [32]. Hence, our results show that optimizing the light intensity can improve the quality of microalgae-derived biodiesel.

### Nitrogen and phosphorus uptake under different light intensities

**Fig. 5:**
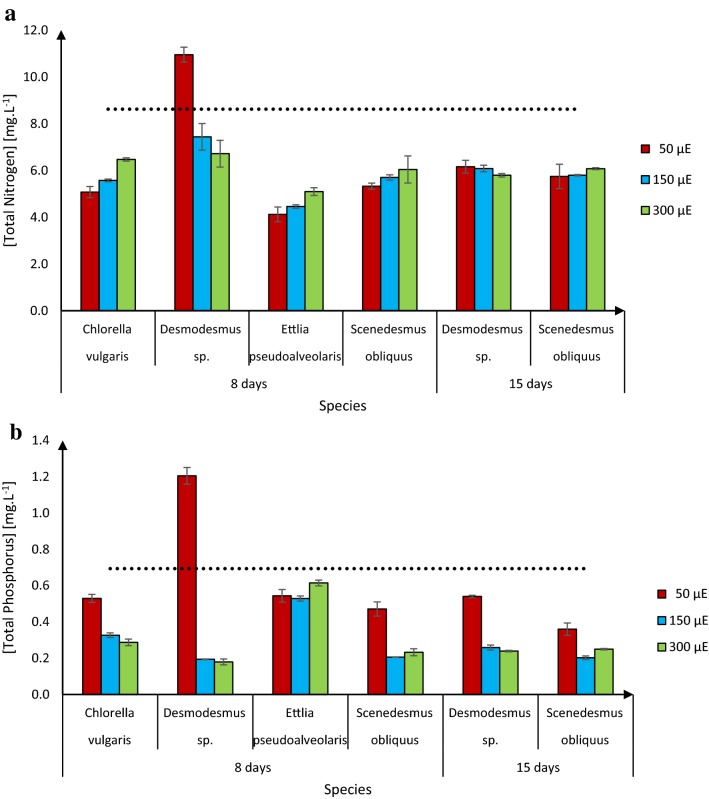
Nitrogen (**a**) and phosphorus (**b**) left in the municipal wastewater used as the growth medium after growth of the four microalgal strains for indicated times under indicated light intensities. Starting values for *N* is 34.5 mg L^−1^ and for P 2.8 mg L^−1^. Dotted lines represent maximum concentrations for release of the wastewater according to the European Directive for Urban Wastewater Treatment. Presented data are means ± standard deviations (*n* = 3 from two separate experiments)

To decrease the cost of biodiesel production, wastewater can be used to supply nutrients for microalgal growth, especially the major nutrients nitrogen and phosphorus [3]. The municipal wastewater used in this study had initial total nitrogen and phosphorus concentrations of 34.5 and 2.8 mg L^−1^, respectively. According to the European Directive for Urban Wastewater Treatment, at least 75% of the total nitrogen and phosphorus should be removed from incoming wastewater before it can be discharged [33]. All the test strains had removed more than 75% of the total nitrogen and phosphorus content of the treated wastewater after 8 days, except *Desmodesmus* sp. at the light intensity of 50 μE (Fig. 5). The amounts of nitrogen and phosphorus removed by *S. obliquus* did not change between 8 and 15 days of cultivation (Fig. 5). Taken together, our results suggest that the microalgae used in this study could take up nitrogen and phosphorus from wastewater, and thus provide a cost-effective wastewater treatment method.

## Conclusions

Our analysis of effects of light on microalgae performed on algae isolated in the Northern hemisphere showed that increases in light intensity increased both biomass and fatty acid contents of two of four tested species (*Desmodesmus* sp. and *S. obliquus*). They also induced changes in fatty acid composition of those species that could improve the quality of biodiesel derived from them. Therefore, *Desmodesmus* sp. and *S. obliquus* seem to be promising candidates for further studies of approaches to optimize biomass and biodiesel production. Another interesting finding, which warrants further mechanistic and physiological attention, is that increases in fatty acid content were accompanied by reductions in protein content.

## Methods

### Algal strains and municipal wastewater

The four algal strains used in this study (*Chlorella vulgaris*, *Desmodesmus sp., Ettlia pseudoalveolaris*, and *Scenedesmus obliquus*) were isolated in Sweden and described by Ferro et al. [34]. Each strain was inoculated and grown in 100 ml of BG11 medium, with a photoperiod of 16 h light (120 μE m^−2^ s^−1^):8 h dark, at 25 °C, and shaking at 150 rpm. Wastewater was provided by the community wastewater plant at Umeå, in Sweden, and stored at − 20 °C. Wastewater was prepared as growth medium by autoclaving after filtration through filter paper with ca. 10 μm pores, provided by Munktell AB (Sweden).

### Algal harvesting and experimental setup

Cultures of each strain were harvested in exponential growth phase by centrifugation (6 min, 3700*g*), washed repeatedly with the treated wastewater medium, and resuspended in it. Initial cultures for the experiments were then prepared by adjusting the optical density (OD_630_) of the suspensions to 0.06 and placing 60 mL aliquots in tubes designed to fit slots in a MC 1000 multi-cultivator (Photon System Instruments, Drásov, Czech Republic). Each species was grown in triplicates at 50, 150 and 300 μE m^−2^ s^−1^ (16 h light: 8 h dark photoperiod). The temperature was 25 ± 2 °C and the tubes were aerated (0.1 L min^−1^). The algae were grown under these conditions for either 8 or 15 days, and OD_630_ was measured daily to monitor their growth. Samples (50 mL) were then harvested by centrifugation at 3700 g for 6 min and freeze-dried for 3 days. Freeze-dried algae were used for lipid extraction and FTIRS analysis. The amounts of nitrogen and phosphorus present in the wastewater were determined before and after each experiment using LCK 138 and LCK349 kits, respectively, and a DR 3900 spectrophotometer, operated according to the manufacturer’s manual (Hach Lange, Germany).

### Lipid extraction

Each freeze-dried sample (2–5 mg) was ground in 5 mL of 4:1 methanol:H_2_O. Then, 4 mL of chloroform was added, the mixture was vortex-mixed, 1.2 mL of 0.73% NaCl solution was added, the mixture was vortex-mixed again and centrifuged at 1250 rpm for 2 min (Wifug, Doctor, Sweden). The lower phase was collected and ¼ of its volume was dried by nitrogen sparging for later use in transmethylation.

### Transmethylation to fatty acid methyl esters (FAMEs)

Dried lipids, prepared as described above, were mixed with 200 μL of a 0.514 mg mL^−1^ solution of pentadecanoic acid, C15:0 (for use as an internal standard) in dry methanol, then 1 mL of 2% H_2_SO_4_ (in dry methanol) was added. After sparging for 2 min, the tubes were immediately closed (to prevent oxygen entering) and heated for 2 h at 80 °C. FAMEs were then extracted from the transmethylation reaction mixture by adding 1 mL of mQ water and 2 mL petrol ether, vortex-mixing and centrifuging at 1250 rpm for 2 min. The top phase was transferred to a new screw-cap tube. This process was repeated using only 2 mL of petroleum ether. The petroleum ether was then sparged with nitrogen gas and dried lipids were finally resuspended in 100 μL of heptane for FAME analysis.

### FAME quantification and composition analysis

A gas chromatograph (GC) equipped with a flame ionization detector (FID) (Thermo scientific Trace 1310) was used to determine FAME content and composition. FAMEs were separated in the GC using a FAME WAX column (30 m × 0.32 mm × 0.25 μm) and the FID signals were analysed using Chromeleon 7.2 software. FAME standards (Sigma Aldrich) used to identify FAMEs in samples were: 14:0 (myristic acid), 16:0 (palmitic acid), 16:1 (palmitoleic acid), 17:0 (margaric acid), 18:0 (stearic acid), 18:1 (oleic acid), 18:2 (linoleic acid), 18:3 (linolenic acid), 20:0 (arachidic acid), and 22:0 (behenic acid). The quantity and composition of FAMEs in the samples were determined from peak areas of identified FAMEs and the internal standard, as previously described [16], using the following formula:

$$ \begin{aligned} & {{{\text{FA content}}}}\left( {\frac{{{\text{mg}}}}{{{\text{g}}}}} \right) = \\ &\frac{1}{100} \frac{{{{{\text{Internal Std. added}}}} \left( {\frac{{{\text{mg}}}}{{{\text{sample}}}}} \right) \frac{{{\text{Area of individual FAME}}}}{{{{{\text{Area of}}}} C15:0 {{{\text{FAME}}}}. {{{\text{Relative Response Factor individual FAME}}}}}}}}{{{\text{Amount of biomass used}}}} \\ \end{aligned} $$

### Fourier transform IR spectroscopy (FTIRS)

The biochemical composition of the algae was examined using FTIRS, as previously described [23, 24], with slight modifications. Briefly, freeze-dried samples and KBr (1:10) were ground and loaded in an IFS 66 FTIR spectrometer equipped with OPUS 6.5 software (Bruker Optik GmbH, Ettlingen, Germany). FTIR spectra were acquired at 400–5200 cm^−1^, signals spanning 800–1850 cm^−1^ were retained, and the baseline was corrected to remove broad background features and keep the low-intensity bands. The relative quantities of carbohydrates, proteins, and lipids were determined by comparing peak intensities at 900–1100 cm^−1^ (carbohydrates), 1738 cm^−1^ (lipids), and 1540 as well as 1658 cm^−1^ (proteins).

## Data Availability

All data generated or analyzed in the current study are included in this article.

## Abbreviations

FTIRS: Fourier-transform IR spectrometry
FAMEs: fatty acid methyl esters
GC: gas chromatograph
FID: flame ionization detector
